# Co-existence of malignant phylloid tumor and metaplastic malignant spindle cell carcinoma: case report

**DOI:** 10.1093/jscr/rjae465

**Published:** 2024-07-25

**Authors:** Ghader Jamjoum, Mariya Alrefaei, Wasan Alhamed, Joud Alsefri, Ahmed Abuzinadah

**Affiliations:** Gastrointestinal Oncology Unit, King Abdulaziz University Hospital, Jeddah 22252, Saudi Arabia; King Abdulaziz University Hospital, Jeddah 22252, Saudi Arabia; King Abdulaziz University Hospital, Jeddah 22252, Saudi Arabia; Fakeeh College for Medical Sciences, Jeddah 21461, Saudi Arabia; Biomedical Department, King Abdulaziz University Hospital, Jeddah, Saudi Arabia

**Keywords:** breast oncology, histopathology, general surgery

## Abstract

Phyllodes tumors (PTs) and Metaplastic Malignant Spindle Cell Carcinoma (MMSCC) are rare and challenging breast malignancies. MMSCC is even rarer and highly aggressive. Surgical excision is the mainstay of treatment for both, but MMSCC generally carries a poorer prognosis. A 46-year-old woman with a history of breast augmentation 4 years ago presented with a rapidly progressing right breast mass. Imaging and core biopsy suggested a malignant PT. A right mastectomy with latissimus dorsi flap reconstruction was done. Initially diagnosed as a malignant PT, the post-operative pathology revealed a co-existence of malignant PT and MMSCC, an extremely rare finding. This case reports the unique challenge of a patient harboring both malignant PT and MMSCC within the same breast tissue. This exceedingly rare co-existence emphasizes the diagnostic complexities associated with uncommon breast malignancies. Our case highlights the challenges of diagnosing and managing uncommon and aggressive breast malignancies.

## Introduction

Phyllodes tumors (PT) are rare breast tumors with both epithelial and stromal parts. Around 75% of them are benign, but breast cancer can also coexist with PTs. Surgical removal with clear margins is recommended for all PTs [1].

Metaplastic breast cancer (MpBC) is an aggressive and rare form of breast cancer. It accounts for only 1% of primary invasive breast carcinomas, and effective management options are limited [2]. One of the subtypes of MpBC is Malignant Spindle Cell Carcinoma (MMSCC) [3]. We report an extremely rare case of a 46-year-old female patient with an unusual histopathology co-existence of malignant PT and MMSCC.

## Case presentation

A 46-year-old woman with a past surgical history of breast augmentation and liposuction noticed a painless, rapidly progressing right breast mass 4 months ago. She has no previous history of breast lumps or biopsies and has one child. She has no family history of breast or ovarian cancer and denied any medical or allergies.

On examination, there was a right breast mass occupying the whole right breast and protruding to the upper outer quadrant. It was attached to the skin, nipple-areola complex, and non-separable from the underlying implant and chest wall muscles. It measured almost 14 cm in the greater diameter. There was no skin erythema or ulcerations. Upon examination, there were no abnormal lymph nodes.

**Figure 1:**
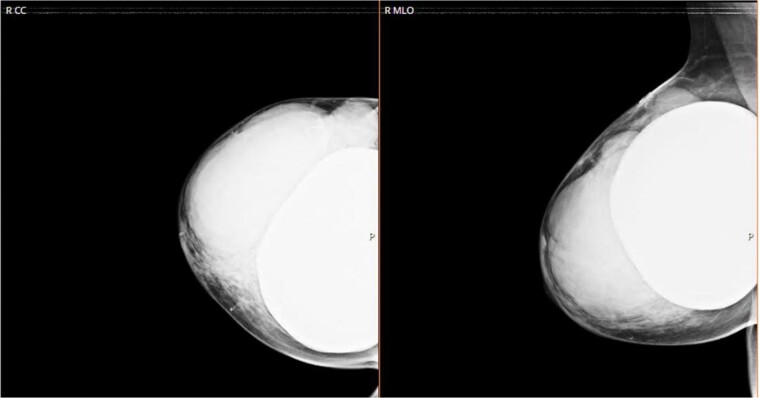
CC and MLO of right breast mammogram.

We sent for a radiological bilateral mammogram craniocaudal and mediolateral oblique, which demonstrated scattered fibro-glandular densities ACR type B in both breasts. Bilateral submammary silicone breast implants show smooth outlines with no free silicone; multiple large lobulated margin masses are replacing most of the right breast parenchyma with no associated calcification seen. The left breast appears unremarkable (Fig. 1).

Complimentary right-sided breast ultrasound showed three circumscribed masses heterogeneous of well-defined contour with cystic changes and hyper flow of interior in color flow Doppler imaging; the largest extends between 11 and 6 o'clock, unremarkable right axillary lymph node.

**Figure 2:**
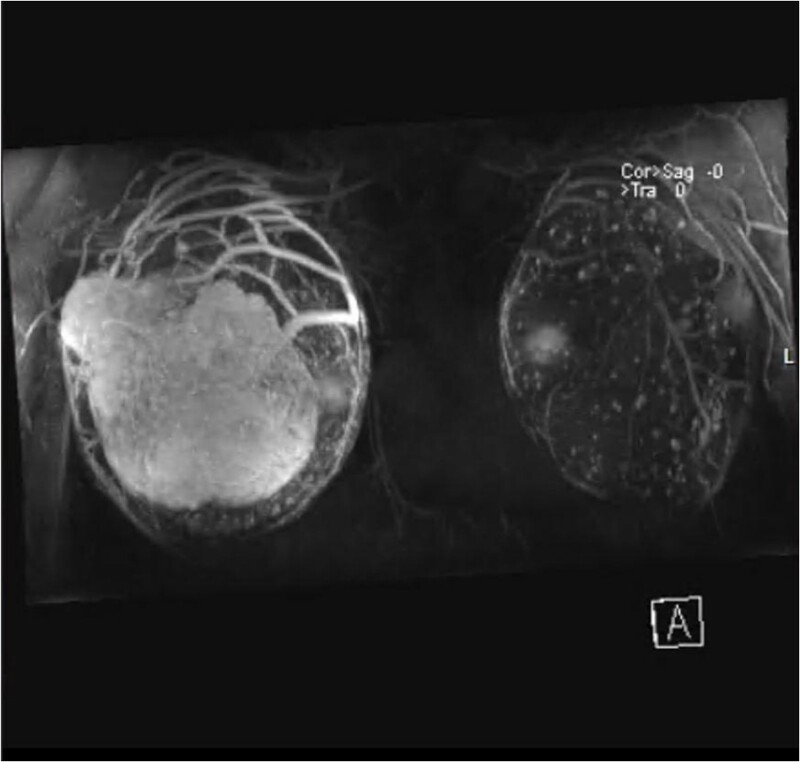
MRI with contrast of right breast and left breast.

Magnetic resonance imaging (MRI) revealed anteriorly two large masses of the right breast, showing heterogeneous intensity and mass effect over the implant. Following contrast injection, peripheral heterogenous enhancement of the well-circumscribed oval mass of the right breast anteriorly occupying the superior lateral aspect measuring 10 cm × 6 cm^2^. Central necrosis is noted. Another smaller mass is seen on the lateral part, which is very close to the skin but with no invasion, measuring 4 cm × 2.6 cm (Fig. 2).

Staging computed tomography of the liver showed homogeneously enhanced, demonstrating a 3-mm hepatic dome simple cyst. There is a right simple ovarian cyst/functional cyst. A focal area of calcification was noted within the myometrium on the right side. The urinary bladder and abdomen were unremarkable. A right breast core needle biopsy showed a malignant spindle cell neoplasm favoring malignant PT. However, MMSCC cannot be excluded with certainty. The patient was diagnosed with a malignant PT in her right breast. After discussion on the breast tumor board, a right total mastectomy with reconstruction of the latissimus dorsi flap was recommended due to the tumor's attachment to the nipples. Despite the recommendation, the patient delayed her decision for two weeks while the tumor continued to grow. The operation was done successfully.

## Extension histopathology and immuno-histochemistry after tumors resection

### Gross

Larger mass: tumor size: 14 × 12 × 8.5 cm^3^—tumor site: superior lateral aspect of the breast. Mitosis: 15/10HPF—atypia: marked—tumor necrosis: present, extensive—no ductal carcinoma in situ is identified. No fibroepithelial elements are identified.

Smaller mass: PT, borderline. Tumor size: 4 × 3.5 × 2.5 cm^3^. Tumor site: superior lateral aspect of the breast. Stromal cellularity: marked. Stromal atypia: moderate to marked. Stromal overgrowth: not identified. Mitotic rate: 7/10PF. Tumor border: focally infiltrative.

### Microscopically

The larger one shows predominantly malignant spindle cell neoplasm with focal areas of epithelioid-like cell morphology; these areas are stained with Ckpan and p63, concerning metaplastic mammary carcinoma. In situ, no epithelial ducts, fibroepithelial components, or ductal carcinoma are found in the malignant spindle cell neoplasm. Based on the histologic features and the presence of an adjacent borderline PT to the larger mass of malignant spindle cell neoplasm, the possibility that this malignant spindle cell neoplasm represents a sarcomatous overgrowth of malignant PT is favored. Despite the fact that there is an apparent focal expression of P63 and CKpan, because of the presence of focal epithelioid-like morphology, higher-than-expected expression of P63 and CKpan in the malignant spindle cell neoplasm, and no direct continuity between the two masses, the possibility of co-existence of PT and metaplastic mammary carcinoma cannot be completely ruled out.

We reviewed this case at our tumor board, and the consensus was to add adjuvant radiation therapy as its very unusual presentation, which carries a high risk of recurrence and poor biology.

## Discussion

MPSCC is rare and poorly studied because of the difficulty in investigating the heterogeneity of metaplastic carcinoma [3].

Henry *et al.* found that all 100 patients with breast cancer presented with a single palpable mass in the upper outer quadrant of the breast [4].

Various patterns are observed in tumor histomorphology [5]. Immunohistochemistry is necessary for considering stromal sarcoma in the differential diagnosis of MPSCCs. Malignant PT can show focal immunoreactivity with antibodies directed against cytokeratins [6]. A core biopsy revealed a malignant spindle cell neoplasm, initially diagnosed as malignant PT. However, the unique histological features suggest that it represents a sarcomatous overgrowth of malignant PT despite the focal apparent expression of P63 and CKpan. Early-stage MMSCC of the breast can be treated with partial mastectomy. Small, potentially early survival benefits can be achieved with radiation. For late-stage disease, complete mastectomy is appropriate, but survival is poor, and radiation has no significant additional benefit [6].

Out of 19 patients, 12 had recurrences after surgery. Lung metastasis was common, and the prognosis was poor due to tumor size and histological grade [7].
